# The Intersection between Spliff Usage, Tobacco Smoking, and Having the First Joint after Waking

**DOI:** 10.1038/s41598-020-64110-4

**Published:** 2020-05-06

**Authors:** Navin Kumar, Cheneal Puljević, Jason Ferris, Adam Winstock, Monica J. Barratt

**Affiliations:** 1 Human Nature Lab, Department of Sociology, Yale University, New Haven, CT USA; 2 School of Public Health, Faculty of Medicine, The University of Queensland, Brisbane, Australia; 3 Centre for Health Services Research, The University of Queensland, Brisbane, Australia; 4 Centre for Health Services Research, The University of Queensland, Woolloongabba, Queensland Australia; 5 University College London, London, UK; 6 Global Drug Survey Ltd, London, UK; 7 Social and Global Studies Centre, RMIT University, Melbourne, Australia; 8 National Drug and Alcohol Research Centre, UNSW Sydney, Sydney, Australia

**Keywords:** Addiction, Epidemiology

## Abstract

Cannabis users who are also tobacco smokers are more likely to exhibit cannabis dependence and psychosocial problems. However, there has been minimal research around various cannabis and tobacco mixing (spliff usage) behaviours and likeliness to smoke the first joint within an hour of waking, known colloquially as wake and bake. The time of first joint and spliff usage may be related as they are associated with the intersection of tobacco and cannabis use. Compared to non-morning cannabis users, morning users reported significantly more cannabis-related problems. Through a survey of US cannabis users, we test the association between various cannabis and spliff use behaviours and likeliness to smoke the first joint within an hour of waking. Compared to those who smoked tobacco and used spliffs, the following spliff use behaviour groups were less likely to have their first joint within 60 minutes after waking: those who smoked tobacco and used spliffs (95%CI: 0.605–0.988); those who never smoked tobacco and did not use spliffs (95%CI: 0.489–0.892); those who never smoked tobacco and used spliffs (95%CI:0.022–0.915). We provided possible explanations for our results and suggested further research to better understand findings, important given expanding US cannabis markets.

## Introduction

The US legal cannabis market has been growing rapidly in recent years, from $2.7 billion in 2014^1^ to $10.4 billion in 2018^2^. Given expanding US cannabis markets, heavy frequent use and cannabis use disorder are a concern^3^. In 2017, in the US, there were approximately 40.9 million people (15.0%) aged 12 or older who used cannabis in the last year^4^. About one in 11 US cannabis users aged 15 or older develops dependent patterns of use, with about 4.2 million people meeting diagnostic criteria for frequent or problematic use^5^. Such patterns of cannabis use are often associated with psychotic symptoms, suicidal ideation and major depressive disorder^6^. These findings are not causal and it is unclear if cannabis is self-medication for mental health conditions. Several studies have detailed US cannabis use prevalence^7–9^, but there has been minimal research around various cannabis and tobacco mixing behaviours and likeliness to smoke the first joint within an hour of waking, known colloquially as wake and bake^10^. The time of first joint and mixing cannabis with tobacco may be related as they are associated with the intersection of tobacco and cannabis use^11,12^. Moreover, cannabis users who are also tobacco smokers are more likely to exhibit cannabis dependence and psychosocial problems^12^. Mixing cannabis with tobacco is defined as the smoking of spliffs-a colloquial term, which are joints (cannabis cigarette) filled with loose-leaf tobacco and cannabis^13^. Our definition does not include blunts, which are partially or completely hollowed out cigar wrappers filled with cannabis^14^. Time to first tobacco cigarette is correlated with many dimensions of nicotine dependence; for example, individuals who smoked their first cigarette within an hour of waking (versus later in the day) tend to smoke more per day and experienced increased difficulty in quitting^15,16^. Similarly, smoking the first cannabis joint within an hour of waking may be a marker of harmful cannabis usage. Compared to non-morning users, morning users reported significantly more cannabis-related problems^10^.

Individuals use spliffs for a variety of reasons, such as; decreased strength and cost of the preparation^17^, and increased uptake of THC^18^. Spliffs are associated with unfavourable consequences, such as forgoing one’s responsibilities or injuring oneself or someone else due to cannabis usage^19^. In addition, the practice exposes users to nicotine^20^, the carcinogenic properties of cigarettes^21^ and respiratory damage^22,23^. As spliff use and time to first joint possibly lead to reduced health consequences, they may be associated. We detail the relationship between the use of spliffs and time to first joint. Cannabis and tobacco use are related and co-use has deleterious effects^24,25^. Thus, we also explore tobacco use and spliff use. Through analysis of a USA-subset of a large cross-sectional online global survey, we test the association between various spliff usage behaviours and likeliness to smoke the first joint within an hour of waking. Findings may provide insight on US cannabis use patterns.

## Methods

### Methods statement

The study received institutional review board (IRB) approval from Kings College London Psychiatry, Nursing and Midwifery Research Ethics Subcommittee (PNM RESC). All research was performed in accordance with relevant guidelines/regulations. All respondents confirmed they were 16+ years and provided informed consent. IRB approval was received to survey those aged ≤18, as per previous studies with the Global Drug Survey (GDS)^26,27^. Similarly, several studies globally did not require parental consent for surveys regarding age groups 16–17^28^.

GDS annually develops and conducts anonymous, online surveys to investigate international trends in drug use, both legal and illicit. US data from GDS 2017, collected from November 15, 2016 to January 18, 2017, is utilized in this paper. Methods utilized here are similar to our previous work with the GDS^9,29^. The survey was actively promoted on social media platforms, such as Twitter, Facebook, and through media partners, such as, Mixmag and The Guardian (USA). We were unable to control for sample variation based on recruitment mode. All respondents confirmed they were 16+ years and provided informed consent. The study received institutional review board (IRB) approval from Kings College London Psychiatry, Nursing and Midwifery Research Ethics Subcommittee (PNM RESC). Survey questions were not forced choice. Responses were included only if individuals indicated use of cannabis in the last 12 months, through all forms of administration, such as smoking, eating and vaporizing. The measures in this paper covered demographic characteristics, whether spliffs were used in the last year, time of first joint, tobacco use, amount of cannabis used per session, number of hours of day spent stoned in a session, time of last joint and number of days cannabis was used in the last year.

Regarding spliff use, participants were asked *When did you last mix cannabis with tobacco?* with the following options: *Never*, *In the last 30 days*, *Between 31 days and 12 months ago*, *More than 12 months ago*. The question referred to loose leaf tobacco being added to cannabis joints (spliffs or cannabis cigarettes), not blunts (cigar wrappers filled with cannabis). The survey item did not explicitly refer to spliff use and this is noted in the Limitations section. Concerning time of first joint, we asked *How soon after you wake up do you smoke your first joint on a day that you use cannabis?* with the following options: *Immediately within 5* *minutes*, *Within less than an hour*, *Within 1*–*4* *hours*, *Within 5*–*12* *hours*, *After more than 12* *hours*. For normal daily cannabis use, we asked participants On a day that you use cannabis how much would you say you normally use? from a dropdown list of 29 weights, starting at 50 mg and gradually increasing to the final selection of >20 g. Regarding the number of hours spent stoned in a session, we asked *On a day that you use cannabis how many hours of the day would you say that you are stoned?* with a dropdown list of 24 options, with the first being 1 Hour with one-hour increments and the last option being 24 Hours. We did not define stoned in the survey, as we assumed the meaning would be apparent to cannabis-using participants. In this paper, we define stoned as any form of cannabis intoxication^30,31^. Regarding tobacco use, we asked *When did you last use Tobacco/cigarettes?* with the options: *Never*, *In the last 30 days*, *Between 31 days and 12 months ago*, *More than 12 months ago*. The survey item refers to tobacco use on its own e.g. someone could answer *Never* but still use spliffs. For the number of days cannabis was used in the year, we asked *During the last 12 months, on how many days have you used cannabis?* and participants keyed in their response. We also asked participants *How long before bed do you have your last joint on a day that you use cannabis?* with the following options: *Last thing before bed*, *1*–*2* *hours before bed*, *3*–*4* *hours before bed*, *More than 4* *hours before bed*. For the *Preferred form of cannabis* item, we provided the following options: *High potency herbal cannabis* (cannabis plant with higher levels of THC), Resin/hash (substance scraped off the cannabis plant and pressed into a lump^32^), *Normal weed/bush/pressed* (lower quality cannabis plant), *Edibles* (food product that contains cannabinoids), *Kief* (cannabis resin sifted from dry cannabis). Regarding the *Most Common Mode of Cannabis Consumption* item, the following were provided: *Oil* (cannabis concentrate consumed by vaporizing), *Butane Hash Oil* (cannabis resin extracted using butane and later vaporized), *Smoked in a joint with tobacco*, *Smoked in a joint without tobacco*, *Smoked in a blunt with tobacco*, *Smoked in a blunt without tobacco*, *Smoked in a pipe with tobacco*, *Smoked in a pipe without tobacco*, *Smoked in a bong/water pipe with tobacco* (bong is a filtration device used for cannabis smoking), *Smoked in a bong/water pipe without tobacco*, *Bucket bong* (a bong variant), *Hot knife* (method of cannabis smoking that uses hot knife blades), *Vaporizer* (a device that generates cannabis in the form of vapor), *Eaten in food*, *Tincture/drank as tea* (cannabis product made by soaking cannabis flowers in ethanol), *Medical spray* (alcohol-based spray containing cannabinoids).

The spliff item was coded as a binary variable: *Never*, *Yes* (Variable A). Time of first joint (cannabis cigarette) in a day was categorized into *>60* *mins* and *<60* *mins* of waking, modelling time to first cigarette^15,33^. Grams of cannabis used per session was recoded into a continuous variable, and the >20 g value was recoded as 21 grams. For ease of interpretation, the age variable was recoded into a categorical variable with breaks of ten years each and consecutive age groups representing less than 5% of the sample were subsumed into a larger group (41–79 years). We created a variable to model patterns of spliff use, coded: *Smoked tobacco in the last 12 months and did not use spliffs*, *Smoked tobacco in the last 12 months and used spliffs*, *Never smoked tobacco and did not use spliffs*, *Never smoked tobacco and used spliffs*, *Smoked tobacco more than 12 months ago and did not use spliffs*, *Smoked tobacco more than 12 months ago and used spliffs* (Variable B). Smoking tobacco refers to smoking tobacco on its own, thus someone could state they never smoked tobacco and still use spliffs.

Logit models, with and without controls, were used to assess if spliff use was associated with time of first joint. We ran two sets of models. We first used spliff use as a binary independent variable (Variable A) and then used various spliff use behaviours as the independent variable (Variable B). We used listwise deletion for missing values. We controlled for demographic characteristics, tobacco use in the last year, time of last cannabis joint, amount of cannabis used per session, number of hours stoned per session and number of days cannabis was used in the last year. We included how long before bed participants had their last joint, as cannabis is sometimes used for sleep initiation and this form of use may contribute to cannabis dependence^34^. Frequency and quantity used per session are associated with problematic cannabis use^35^. We included number of days cannabis was used in the last 12 months and amount of cannabis used per session. Time spent intoxicated on cannabis is a marker of problematic use^36^ and we included a measure of number of hours spent stoned in a session.

Odds ratios (ORs), adjusted odds ratios (aORs), 95% confidence intervals (95% CI) and p-values were reported. Participants were not required to answer every question, resulting in some missing data. Given the structured sequence of questions, missing values for *mixing tobacco with cannabis* meant that participants had not used a spliff e.g. if someone indicated they did not use cannabis, the mixing tobacco with cannabis (spliff use) value would be missing. Thus, we coded missing values for *mixing tobacco with cannabis* (spliff use) as *Never*. When accounting for questions participants were not required to answer, the percentage of missing values for all variables did not exceed 20% (see Supplementary Table I). Multiple imputation was utilized to appropriately handle missing values, assumed to be missing at random. Given the number of categorical variables, we used the predictive mean matching technique^37^ with only the variables described in this study. Similar analyses were run on the imputed dataset (see Supplementary Table II). We generated 10 imputation data sets. Statistical tests were performed on original and imputed data sets to determine the extent of result convergence^38^. All analysis was conducted using R with the following packages: *dplyr, stargazer, plyr, lmtest, multiwayvcov, sandwich, mice*^37,39–46^.

## Results

### Sample

From November 2016 to January 2017, a total of 10183 responses were recorded in the US. 8345 (82%) participants reported cannabis use in the past year. A further 1955 records were excluded due to missing data. The remaining 6390 respondents formed the sample for analysis. Males accounted for 75.48% of the sample, with a median age of 23 (interquartile range (IQR): 19–32, Range: 16–79, see Table 1).

**Table 1:** Variables of Interest (Number of Participants Reporting Cannabis Use in Last Year = 8345).

Age (N = 8345)	-16–20	28.4%
	-21–30	41.7%
	-31–40	14.9%
	-41–79	15.0%
Sex (N = 8345)	-Male	75.5%
	-Female	23.6%
	-Transgender	0.9%
Time of First Joint (N = 7033)	>60 mins	78.0%
	<60 mins	22.0%
Time of Last Joint (N = 7034)	-Last Thing before Bed	31.3%
	-1-2 hours before bed	49.1%
	-3-4 hours before bed	15.4%
	-More than 4 hours before bed	4.2%
Spliff Use (N = 8345)	No	78.0%
	Yes	22.0%
Cannabis Used Per Session (Grams) (N = 7667)	Median	0.5
	Interquartile Range	0.125–1.000
Number of Hours Stoned in a Session (N = 6970)	Median	4
	Interquartile Range	3.0–6.0
Number of Hours Stoned in a Session for those who had their Last Joint Just Before Bed (N = 1926)	Median	6
	Interquartile Range	3.0–11.0
Number of Days Cannabis was Used in the Last Year (N = 7389)	Median	250
	Interquartile Range	50–360
Preferred Form of Cannabis (N = 7565)	-High potency herbal cannabis	62.1%
	-Resin/hash	11.2%
	-Normal weed/bush/pressed	1.7%
	-Edibles	1.3%
	-Kief	8.3%
	-Oil	8.0%
	-Butane Hash Oil	7.6%
Most Common Mode of Cannabis Consumption (N = 7913)	-Smoked in a joint with tobacco	3.8%
	-Smoked in a joint without tobacco	11.3%
	-Smoked in a blunt with tobacco	0.7%
	-Smoked in a blunt without tobacco	7.5%
	-Smoked in a pipe with tobacco	0.5%
	-Smoked in a pipe without tobacco	33.3%
	-Smoked in a bong/water pipe with tobacco	2.6%
	-Smoked in a bong/water pipe without tobacco	23.0%
	-Bucket bong	1.5%
	-Hot knife	0.2%
	-Vaporizer	12.7%
	-Eaten in food	2.4%
	-Tincture/drank as tea	0.4%
	-Medical spray	0.1%

### Demographic characteristics

Of those who reported cannabis use in the past year, most (78%) reported consuming their first joint more than an hour after waking and most (78%) tended not to use spliffs (see Table 1). Participants used cannabis for a median of 250 days in the last year (almost daily), with 0.50 grams the median for use per session. Participants spent a median of four hours a day stoned when cannabis was used. Those who had their last joint just before bed spent a median of six hours a day stoned. About half the sample had their last joint 1–2 hours before bed. When comparing cannabis use behaviours across spliff use groups, there were clear differences in gender distributions (see Table 2). For those who smoke tobacco and used spliffs, and never smoke tobacco and used spliffs, about half were in the 16–20 age group. For all other groups and the broader sample, the 16–20 group was a relatively small proportion. Distributions across all other variables were relatively similar across groups and mirrored the broader sample.

**Table 2:** Differences in Cannabis Use Behaviors Across Tobacco and Spliff Usage Groups.

Variable	Level	Smoke tobacco and did not use spliffs (n = 2008)	Smoke tobacco and used spliffs (n = 1870)	Never smoked tobacco and did not use spliffs (n = 3097)	Never smoked tobacco and used spliffs (n = 250)	Smoked tobacco more than 12 months ago and did not use spliffs (n = 963)	Smoked tobacco more than 12 months ago and used spliffs (n = 157)	Differences in means (P-Value)
Age	-16–20	20.17%	40.37%	31.74%	51.20%	7.17%	17.83%	*p* = 0.00
	-21–30	44.32%	48.82%	38.68%	40.80%	30.94%	52.23%	
	-31–40	20.27%	8.40%	14.01%	5.60%	21.29%	17.83%	
	-41–79	15.24%	2.41%	15.56%	2.40%	40.60%	12.10%	
Sex	-Male	72.26%	79.04%	75.65%	84.40%	73.42%	69.43%	*p* = 0.00
	-Female	26.74%	20.05%	23.47%	15.60%	25.65%	30.57%	
	-Transgender	1.00%	0.91%	0.87%	—	0.93%	—	
Time of First Joint	>60 mins	74.71%	75.94%	83.37%	83.13%	77.39%	75.66%	*p* = 0.00
	<60 mins	25.29%	24.06%	16.63%	16.87%	22.61%	24.34%	
Time of Last Joint	-Last Thing before Bed	33.06%	32.53%	26.07%	29.32%	34.98%	37.50%	*p* = 0.00
	-1-2 hours before bed	48.25%	51.24%	48.30%	49.40%	47.73%	50.00%	
	-3-4 hours before bed	14.08%	13.82%	19.15%	18.07%	14.12%	10.53%	
	-More than 4 hours before bed	4.61%	2.42%	6.49%	3.21%	3.16%	1.97%	
Cannabis Used Per Session (Grams)	Median	0.5	0.5	0.5	0.5	0.5	0.5	*p* = 0.00
	Interquartile Range	0.10–1.00	0.25–1.50	0.10–1.00	0.25–1.50	0.10–1.00	0.1875–1.000	
Number of Hours Stoned in a Session	Median	4	5	4	4	4	4	*p* = 0.00
	Interquartile Range	3.00–6.00	3.00–7.00	3.0–6.0	3.0–6.0	3.0–8.0	3.00–8.00	
Number of Days Cannabis was Used in the Last Year	Median	250	250	200	240	300	300	*p* = 0.00
	Interquartile Range	36.0–360.0	100.0–350.0	30.0–350.0	75.0–340.0	54.0–365.0	100.0–350.0	
Number of Hours Stoned in a Session for those who had their Last Joint Just Before Bed	Median	6	6	5	6	6	6	*p* = 0.17
	Interquartile Range	3–10	4–10	3–10	3–12	3–11	4–12	

### Consuming first joint within an hour after waking and spliff use

**Figure 1:**
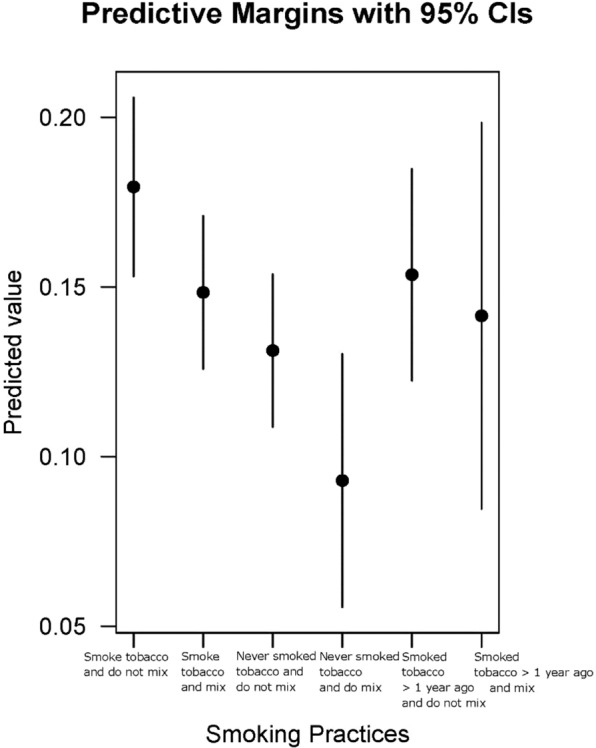
Predicted probability graph. Predicted probability graph for differences in tobacco use and spliff usage patterns.

No statistically significant associations were found for the logistic regression with or without controls, for the relationship between spliff use (*Never, Yes*) and smoking the first joint within an hour after waking. Table 3 indicates the results of the logistic regression, with and without controls, regarding consuming the first joint within an hour after waking, and various spliff use patterns. When accounting for controls, compared to those who smoked tobacco and did not use spliffs, the following groups were less likely to have their first joint within 60 minutes after waking: those who smoked tobacco and did use spliffs (aOR = 0.80, p = 0.02), those who never smoked tobacco and did not use spliffs (aOR = 0.69, p = 0.00), those who never smoked tobacco and did use spliffs (aOR = 0.47, p = 0.00). Those who never smoked tobacco and did use spliffs had the lowest probability of smoking within the first hour and those who smoked tobacco but did not use spliffs had the highest probability (see Fig. 1). Results of logistic regression analysis run on the original and imputed data converged for all explanatory variables and categories (see Supplementary Table II).

**Table 3:** Consuming First Joint Within an Hour after Waking and Spliff Usage Patterns, With and Without Adjusting for Controls (N = 6390).

Variable	Level	*n*	OR (95% CI)	P-value	aOR (95% CI)	P-value
Spliff Usage Groups	Smoke tobacco and did not use spliffs	1802	1.00	—	1.00	—
	Smoke tobacco and used spliffs	1699	0.93 (0.78, 1.09)	0.38	0.80 (0.61, 0.99)**	0.02
	Never smoked tobacco and did not use spliffs	1646	0.60 (0.43, 0.77)***	0.00	0.69 (0.49, 0.89)***	0.00
	Never smoked tobacco and used spliffs	228	0.57 (0.20, 0.94)***	0.00	0.47 (0.02, 0.92)***	0.00
	Smoked tobacco more than 12 months ago and did not use spliffs	877	0.88 (0.69, 1.07)	0.17	0.83 (0.60, 1.06)	0.12
	Smoked tobacco more than 12 months ago and used spliffs	138	0.96 (0.56, 1.36)	0.85	0.75 (0.28, 1.23)	0.24
Age	16–20	1756	—	—	1.00	—
	21–30	2692	—	—	1.08 (0.89, 1.26)	0.44
	31–40	979	—	—	1.08 (0.84, 1.32)	0.53
	41–79	963	—	—	0.61 (0.35, 0.87)***	0.00
Sex	Male	4864	—	—	1.00	—
	Female	1470	—	—	1.07 (0.90, 1.24)	0.45
	Transgender	56	—	—	1.50 (0.76, 2.24)	0.28
Cannabis Used Per Session	—	6390	—	—	1.17 (1.12, 1.21)***	0.00
Number of Hours Stoned in a Session	—	6390	—	—	1.19 (1.17, 1.20)***	0.00
Number of Days Cannabis was Used in the Last Year	—	6390	—	—	1.00 (1.00, 1.00)***	0.00
Time of Last Joint	Last Thing before Bed	2006	—	—	1.00	—
	1–2 hours before bed	3137	—	—	0.46 (0.31, 0.60)***	0.00
	3–4 hours before bed	984	—	—	0.15 (−0.21, 0.52)***	0.00
	More than 4 hours before bed	263	—	—	0.24 (−0.43, 0.90)***	0.00
Constant	—	—	0.34 (0.23, 0.45)	—	0.06 (−0.23, 0.35)	—
*N*			6390	—	6390	—

^a^OR, adjusted odds ratio. OR, odds ratio. **p < 0.05; ***p < 0.01.

## Discussion

We sought to test the association between various spliff usage behaviours and likeliness to smoke the first joint within an hour of waking, among US cannabis users. Accounting for controls, compared to those who smoked tobacco and did not use spliffs, the following were less likely to have their first joint within an hour after waking: those who smoked tobacco and used spliffs, those who never smoked tobacco and did not use spliffs, those who never smoked tobacco and used spliffs.

There are a range of explanations for our results. We explore two possible explanations, in line with the limitations of our data. Harm reduction, of a life-functioning variant instead of a pulmonary/respiratory variant^47^, may explain why respondents who smoked tobacco and used spliffs, did not want to have their first joint within an hour of waking. Some participants may be aware of the harms of tobacco smoking combined with spliff use and thus seek to reduce engagement in another harmful behaviour; waking and baking. Another explanation may be demographic differences across spliff usage groups. We indicate proportionately more younger participants in certain spliff usage groups, possibly related to our outcome variable. Similarly, cannabis and tobacco users have differing outcomes compared to those who use only cannabis^12^. Qualitative research around specific demographic groups and tobacco and cannabis use behaviours may provide further insight.

### Limitations

We conducted likely the largest US study testing the association between various spliff usage behaviours and having the first joint within an hour after waking. This research design has costs and benefits, such as population level^29,48,49^ reliability and validity. When data are limited, online surveys may be valid. Comparable probability sampling and ethnographic data may be key to increase validity of our findings^50^. The age and sex distributions of cannabis users who completed the GDS were similar to demographic distributions in a household survey across Australia, the US, and Switzerland^29^. The GDS is therefore an efficient means of getting a gradated insight around stigmatised behaviours as long as the survey is not used to determine population-level drug prevalence^29^.

We did not have information on how nicotine dependence may have influenced the results; frequency of tobacco use and time to first tobacco cigarette. This information may help explain our findings and future research can incorporate these survey items. As we used an online survey of US drug users, our sample was skewed toward younger participants. We used age and sex as controls, but other covariates such as sexuality, urban/non-urban residence and recruitment mode were not included. Not everyone in the sample reported smoking joints and future studies can exclusively recruit joint smokers. Our definition of spliffs did not include blunts, which can be detailed in future research. The survey item did not explicitly refer to spliffs and it is possible that some respondents thought the item indicated other ways of mixing cannabis with tobacco. We will explicitly refer to spliffs in future survey iterations. It is possible that participants who reported not smoking tobacco but use spliffs mistakenly reported consuming tobacco on one question but not the other. We were not able to control for such effects but will include corrective mechanisms in future survey iterations. Those who had their last joint just before bed had a greater median time spent stoned compared to the larger sample. We did not conduct analysis with time of last joint, which may be marker of problematic use. We handled missing data with multiple imputation. While results from original and imputed datasets converged, a reduced rate of missing data would increase reliability of findings.

### Concluding statement

Accounting for controls, in the US, compared to those who smoked tobacco and did not use spliffs, those with the following spliff usage behaviours were less likely to have their first joint within 60 minutes of waking: those who smoked tobacco and used spliffs; those who never smoked tobacco and did not use spliffs; those who never smoked tobacco and used spliffs. We provided some possible explanations for our results and suggested further research to better understand findings. Overall, we shed light on time of first joint and spliff usage behaviours, important given expanding US cannabis markets.

## Supplementary information

Supplementary information.

## References

[1] The Arcview Group*. The State of Legal Marijuana Markets - 3rd Edition*, https://arcviewgroup.com/product/6th-edition/ (2015).

[2] Reisinger, D. The Legal Marijuana Industry Is Soaring—And 2019 Could Be Its Best Year Yet. *Fortune*, http://fortune.com/2018/12/27/legal-marijuana-industry-sales/ (2018).

[3] Blanco C (2016). Cannabis use and risk of psychiatric disorders: prospective evidence from a US national longitudinal study. *JAMA*. Psychiatry.

[4] Center for Behavioral Health Statistics and Quality. *2017 National Survey on Drug Use and Health: Detailed Tables*. (2018).

[5] Han, B., Hedden, S., Lipari, R., Copello, E. & Kroutil, L. Receipt of services for behavioral health problems: results from the 2014 National Survey on Drug Use and Health. *Rockv. MD Subst. Abuse Ment. Health Serv. Adm*. (2015). 29431966

[6] Fergusson, D. M. & Boden, J. M. Cannabis use and later life outcomes. *Addict. Abingdon Engl*. **103**, 969–976, discussion 977–978 (2008). 10.1111/j.1360-0443.2008.02221.x 18482420

[7] Hasin DS (2016). Prevalence and correlates of DSM-5 cannabis use disorder, 2012–2013: Findings from the National Epidemiologic Survey on Alcohol and Related Conditions–III. Am. J. Psychiatry.

[8] Hasin DS (2015). Prevalence of Marijuana Use Disorders in the United States Between 2001–2002 and 2012–2013. JAMA Psychiatry.

[9] Kumar N, Puljević C, Ferris J, Winstock A, Barratt MJ (2019). Cannabis use patterns at the dawn of US cannabis reform. J. Cannabis Res.

[10] Earleywine M, Luba R, Slavin MN, Farmer S, Loflin M (2016). Don’t wake and bake: morning use predicts cannabis problems. Addict. Res. Theory.

[11] Agrawal A, Budney AJ, Lynskey MT (2012). The co-occurring use and misuse of cannabis and tobacco: a review: Cannabis and tobacco review. Addiction.

[12] Peters EN, Peters EN, Budney AJ, Carroll KM (2012). Clinical correlates of co-occurring cannabis and tobacco use: a systematic review. Addiction.

[13] Williams, K. What’s the difference between joints, blunts, and spliffs? *Leafly*, https://www.leafly.com/news/cannabis-101/whats-the-difference-between-joints-blunts-and-spliffs (2015).

[14] Soldz S, Huyser DJ, Dorsey E (2003). The cigar as a drug delivery device: youth use of blunts. Addiction.

[15] Baker TB (2007). Time to first cigarette in the morning as an index of ability to quit smoking: implications for nicotine dependence. Nicotine Tob. Res..

[16] Toll BA, Schepis TS, O’Malley SS, McKee SA, Krishnan-Sarin S (2007). Subjective reactivity to the first cigarette of the day as a predictor of smoking relapse: a preliminary study. Drug Alcohol Depend.

[17] Akre C, Michaud P-A, Berchtold A, Suris J-C (2009). Cannabis and tobacco use: where are the boundaries? A qualitative study on cannabis consumption modes among adolescents. Health Educ. Res..

[18] Van der Kooy F, Pomahacova B, Verpoorte R (2009). Cannabis smoke condensate II: influence of tobacco on tetrahydrocannabinol levels. Inhal. Toxicol..

[19] Baggio S (2014). Routes of Administration of Cannabis Used for Nonmedical Purposes and Associations With Patterns of Drug Use. J. Adolesc. Health.

[20] Dunlap E, Benoit E, Sifaneck SJ, Johnson BD (2006). Social constructions of dependency by blunts smokers: Qualitative reports. Int. J. Drug Policy.

[21] van Beurden EK, Zask A, Passey M, Kia AM (2008). The Mull Hypothesis: is cannabis use contributing to high tobacco use prevalence among young North Coast males?. New South Wales Public Health Bull.

[22] Copeland, J. & Alperstein, D. Cannabis: Use, harms, disorder, and interventions. *Aust. Clin. Psychol*. **2** (2016).

[23] Macleod J (2015). Cannabis, tobacco smoking, and lung function: a cross-sectional observational study in a general practice population. Br J Gen Pr..

[24] Schauer GL, Berg CJ, Kegler MC, Donovan DM, Windle M (2015). Assessing the overlap between tobacco and marijuana: Trends in patterns of co-use of tobacco and marijuana in adults from 2003–2012. Addict. Behav..

[25] Schauer GL, King BA, McAfee TA (2017). Prevalence, correlates, and trends in tobacco use and cessation among current, former, and never adult marijuana users with a history of tobacco use, 2005–2014. Addict. Behav..

[26] Demant, D. *et al*. Differences in substance use between sexual orientations in a multi-country sample: findings from the Global Drug Survey 2015. *J. Public Health* jphm;fdw069v2, 10.1093/pubmed/fdw069 (2016). 10.1093/pubmed/fdw069 27519959

[27] Hughes CE, Barratt MJ, Ferris JA, Maier LJ, Winstock AR (2018). Drug-related police encounters across the globe: How do they compare?. Int. J. Drug Policy.

[28] Helweg-Larsen K, Bøving-Larsen H (2003). Ethical issues in youth surveys: Potentials for conducting a national questionnaire study on adolescent schoolchildren’s sexual experiences with adults. Am. J. Public Health.

[29] Barratt MJ (2017). Moving on from representativeness: Testing the utility of the Global Drug Survey. Subst. Abuse Res. Treat.

[30] Green B, Kavanagh D, Young R (2003). Being stoned: a review of self‐reported cannabis effects. Drug Alcohol Rev.

[31] Tart, C. T. & Stoned, O. B. A Psychological Study of Marijuana Intoxication. *Palo Alto Sci. Behav. Books* (1971).

[32] Ascert. Cannabis Resin|Ascert, https://www.ascert.biz/drug-and-alcohol-information/a-z-drugs/cannabis-resin/.

[33] Haberstick BC (2007). Genes, time to first cigarette and nicotine dependence in a general population sample of young adults. Addiction.

[34] Babson KA, Bonn-Miller MO (2014). Sleep Disturbances: Implications for Cannabis Use, Cannabis Use Cessation, and Cannabis Use Treatment. Curr. Addict. Rep.

[35] Asbridge M, Duff C, Marsh DC, Erickson PG (2014). Problems with the Identification of ‘Problematic” Cannabis Use: Examining the Issues of Frequency, Quantity, and Drug Use Environment’. Eur. Addict. Res..

[36] Bonn-Miller MO, Heinz AJ, Smith EV, Bruno R, Adamson S (2016). Preliminary Development of a Brief Cannabis Use Disorder Screening Tool: The Cannabis Use Disorder Identification Test Short-Form. Cannabis Cannabinoid Res.

[37] Buuren Svan, Groothuis-Oudshoorn K (2011). mice: Multivariate Imputation by Chained Equations in R. J. Stat. Softw..

[38] Origer A, Le Bihan E, Baumann M (2014). Social and economic inequalities in fatal opioid and cocaine related overdoses in Luxembourg: A case–control study. Int. J. Drug Policy.

[39] Graham, N., Arai, M., Hagströmer, B. & Graham, M. N. *Package ‘multiwayvcov’*. (2016).

[40] Hlavac, M. *Stargazer: Well-Formatted Regression and Summary Statistics Tables*. (Harvard University, 2015).

[41] R Core Team. *R: A Language and Environment for Statistical Computing*. (R Foundation for Statistical Computing, 2018).

[42] Wickham, H. *The Split-Apply-Combine Strategy for Data Analysis*. (2011).

[43] Wickham, H., Francois, R., Henry, L. & Müller, K. *dplyr: A Grammar of Data Manipulation* (2017).

[44] Zeileis A (2004). Econometric Computing with HC and HAC Covariance Matrix Estimators. J. Stat. Softw..

[45] Zeileis A (2006). Object-Oriented Computation of Sandwich Estimators. J. Stat. Softw..

[46] Zeileis, A. & Hothorn, T. *Diagnostic Checking in Regression Relationships* (2002).

[47] Karoll BR (2010). Applying social work approaches, harm reduction, and practice wisdom to better serve those with alcohol and drug use disorders. J. Soc. Work.

[48] Winstock AR (2011). Mephedrone, new kid for the chop?. Addiction.

[49] Winstock AR, Barratt MJ (2013). Synthetic cannabis: a comparison of patterns of use and effect profile with natural cannabis in a large global sample. Drug Alcohol Depend.

[50] Barratt MJ, Ferris JA, Lenton S (2015). Hidden populations, online purposive sampling, and external validity: Taking off the blindfold. Field Methods.

