# Author Correction: The intersection between spliff usage, tobacco smoking, and having the first joint after waking

**DOI:** 10.1038/s41598-020-67809-6

**Published:** 2020-07-06

**Authors:** Navin Kumar, Cheneal Puljević, Jason Ferris, Adam Winstock, Monica J. Barratt

**Affiliations:** 1 Human Nature Lab, Department of Sociology, Yale University, New Haven, CT USA; 2 School of Public Health, Faculty of Medicine, The University of Queensland, Brisbane, Australia; 3 Centre for Health Services Research, The University of Queensland, Brisbane, Australia; 4 Centre for Health Services Research, The University of Queensland, Woolloongabba, QLD Australia; 5 University College London, London, UK; 6 Global Drug Survey Ltd, London, UK; 7 Social and Global Studies Centre, RMIT University, Melbourne, Australia; 8 National Drug and Alcohol Research Centre, UNSW Sydney, Sydney, Australia

Correction to: *Scientific Reports* 10.1038/s41598-020-64110-4, published online 06 May 2020

The original version of this Article contained typographical errors in the Abstract.

“Compared to those who smoked tobacco and used spliffs, the following spliff use behaviour groups were less likely to have their first joint within 60 minutes after waking: those who smoked tobacco and used spliffs (95%CI: 0.605–0.988); those who never smoked tobacco and did not use spliffs (95%CI: 0.489–0.892); those who never smoked tobacco and used spliffs (95%CI:0.022–0.915).”

now reads:

“Compared to those who smoked tobacco and did not use spliffs, the following spliff use behaviour groups were less likely to have their first joint within 60 minutes after waking: those who smoked tobacco and used spliffs (95%CI: 0.605–0.988); those who never smoked tobacco and did not use spliffs (95%CI: 0.489–0.892); those who never smoked tobacco and used spliffs (95%CI:0.022–0.915).”

This has now been corrected in the PDF and HTML versions of the Article.

